# The Influence of Gender on Choosing Ophthalmology as a Career Among Medical Students and Interns in Madinah, Saudi Arabia

**DOI:** 10.7759/cureus.44936

**Published:** 2023-09-09

**Authors:** Ghada A Aljuhani, Mona Abdulaziz, Abeer S Alharbi

**Affiliations:** 1 Ophthalmology, Saudi Commission for Health Specialties, Madinah, SAU; 2 Ophthalmology, Ohud Hospital, Medina, SAU

**Keywords:** influence, gender, factors, career, ophthalmology

## Abstract

Introduction

The field of ophthalmology has become increasingly popular among medical students and interns in recent years. However, there may be gender-based differences in the factors influencing the choice of ophthalmology as a career path. This study aimed to investigate the influence of gender on the decision to pursue ophthalmology as a career among medical students and interns in Madinah, Saudi Arabia.

Methods

This cross-sectional study involved medical students and interns in Madinah, Saudi Arabia. A self-administered questionnaire was used to collect data from the participants at two medical colleges from March to May 2023. The questionnaire examined potential factors that would influence the students to choose or eliminate ophthalmology as a future career and whether there is a gender difference.

Result

A total of 449 medical students were included. Two hundred eighteen were males, and 231 were females. The mean age was 22; around (30%) of the participants were in the fourth year of medical school, and most respondents (63%) had no prior exposure to ophthalmology. Fewer working hours was the driving factor among the males to choose ophthalmology as a speciality (58%); in contrast to females, interest in eye anatomy and physiology was the main factor (60%). Being already passionate about other specialties was the main factor that deterred the students from choosing ophthalmology as a career among both males and females, with a p-value of 0.033.

Conclusion

Our study has shown that gender plays no significant role in influencing medical students' choice to pursue ophthalmology as a future career. Teaching students in early medical years about ophthalmology as an option may lead to significant contributions to understanding and determining their future path.

## Introduction

Choosing a career path is not an easy process, and it may be one of the most challenging decisions a medical graduate can make. Ophthalmology is one of the exciting specialties to pursue a career in. It combines both medical and surgical aspects [1]; it is one of the most desired potential career paths for medical students. [2,3]. Since more efforts are taking place in Saudi Arabia to improve women's power in all aspects of education and employment [4], medicine is one of these careers, which is in need of gender equality and woman empowerment.

According to a previous study, there was a gender difference for most of the specialties, and surgical options were among them [5].

One of the examples of gender differences in the field of ophthalmology internationally is that female ophthalmologists are more prone to challenges and discrimination in their careers compared to males [6]. One study in the United States has shown gender differences in writing recommendation letters for ophthalmology applicants with more bias toward male recommendations [7].

Multiple studies have investigated the different factors that may influence medical students to choose ophthalmology as a career in Saudi Arabia; however, none of them assessed the role of gender in ophthalmology considerations [8,9].

This is the first study focusing on gender bias in choosing ophthalmology as a specialty. Knowing the factors that influence medical students and interns to choose or eliminate ophthalmology and whether there is a gender difference will help get an overview of misconceptions and take early action to correct them.

## Materials and methods

This was a cross-sectional study conducted by using an electronic questionnaire distributed through WhatsApp to the online groups of medical students of Taibah University and Alrayan College in Madinah, Saudi Arabia. Across all levels, during the period from March 2023 to April 2023. All medical students and interns from these universities were included; we excluded graduates and students in the preparatory year.

The questionnaire involved the following parts: demographic data, the part about prior experience in ophthalmology, the desire to pursue a career in ophthalmology, factors influencing the choice of pursuing a career in ophthalmology, and factors affecting the elimination of ophthalmology as a career.

Previous ophthalmology experience included the following options: none, core rotations, elective rotations, ophthalmology mentor, ophthalmologist family member, and attended an ophthalmology conference.

The research protocol adhered to the principles of the World Medical Association Declaration of Helsinki, and the institutional review board registered the study at Madinah Health Affairs. Informed consent was taken from all participants at the beginning of the questionnaire.

A pilot study of 30 participants was completed before the distribution of the questionnaire, and it was removed from the final sample size.

Data were extracted to an Excel sheet (version 16.6; Microsoft, Redmond, Washington), and then SPSS version 29 (IBM Inc., Armonk, New York) was used for data analysis, the Chi-squared test was used, and a p-value of <0.005 was considered significant. 

## Results

The total sample size was 449; 218 (49.6%) were males, and 231 (51.4%) were females. The mean age was around 22, with 18 as the minimum and 29 as the maximum. Ninety (41%) of the males were considering ophthalmology as a future career, with a similar ratio of the females 86 (37%; Table 1), and there were no significant relationships between the two groups (p=0.379). As shown in (Table 2), most students were in the fourth year of medical school. Most of our respondents (63%) had no prior exposure to ophthalmology, which was a similar finding in both genders. 

**Table 1:** Previous exposure to ophthalmology

Previous exposure to ophthalmology	Male	Female	p-value
Yes	74	90	0.270
No	144	141	

**Table 2:** Level of medical students

Level of medical students	Number	%
First year	74	16.5
Second year	74	16.5
Third year	76	16.9
Fourth year	103	22.9
Fifth year	68	15.1
Internship	54	12.0

Regarding the factors that influenced the choice to pursue ophthalmology (Table 3), we found the leading factor to be fewer working hours for the males and potential for a good work-life balance for the females whatsoever there was no significant difference between males and females when choosing ophthalmology as a future specialty as p-value were above 0.05.

**Table 3:** The factors influencing the choice of ophthalmology as a future career based on gender

Factor	Male (n)	Female (n)	p-value
Interest in eye anatomy and physiology	50	52	0.914
Exposure to Ophthalmology during medical school	28	29	0.927
Mentorship or guidance from an ophthalmologist	15	17	0.844
Potential for work-life balance	47	59	0.321
High income	48	50	0.924
Fewer working hours	53	48	0.370
Visual learner	17	29	0.097
Available job opportunities	23	24	0.956
Direct patient contact	26	22	.410
Impact on patient care	26	29	.839
Perception of the specialty by others	10	15	.379
Job satisfaction	40	40	.755
Length of residency training (fewer years of training)	23	23	.836
Positive training experience	18	20	.879
Positive research experience	13	11	.572

The perception of the specialty by others was the least to consider when choosing ophthalmology among males, while positive research experience was the least influential factor to consider among females.

Already being passionate about other specialties was the main factor that led to eliminating ophthalmology as a career (Table 4) among male and female medical students. 

**Table 4:** The factors deterring medical students from pursuing ophthalmology as a future career based on gender

Factor	Male (n)	Female (n)	p-value
Lack of interest in eye anatomy and physiology	39	70	0.002
Lack of exposure to ophthalmology during medical school	29	56	0.003
Lack of mentorship or guidance from an ophthalmologist	18	21	0.754
Perception of poor work-life balance	6	8	0.665
Direct patient contact	6	13	0.130
highly competitive specialty	30	48	0.050
Already passionate about another specialty	94	77	0.033
Perception of lack of job satisfaction	14	10	0.324
Perception of lack of job opportunities	17	18	0.998
Negative personal experience in ophthalmology	15	8	0.101
Perception as a male-dominated specialty	1	5	0.116
Lack of research opportunities	5	5	0.926

The choices, lack of interest in eye anatomy and physiology (p-value=0.002), lack of exposure to ophthalmology during medical school (p-value=0.003), highly competitive specialty (p-value=0.050), and already being passionate about another specialty (p-value=0.033) showed a significant relationship between males and females otherwise there was no significant relationship between the two genders. 

## Discussion

This is a survey-based cross-sectional study to evaluate any gender-related differences in relation to pursuing ophthalmology as a potential career between medical students and interns in Madinah, Saudi Arabia. To our knowledge, this is the first study to highlight the effect of gender on factors influencing the student's choice of future specialty.

The total sample size was 449, where (49%) were males and (51%) were females. While there were slightly more female participants (n=231) compared to males (n=218), most of both genders expressed interest in ophthalmology as a potential specialty for residency training. Nearly two-thirds of participants from both genders had no previous ophthalmology experience or exposure, indicating their interest was not based on prior background. 

These findings are consistent with the recent trends of increasing numbers of women entering surgical specialties like ophthalmology [10,11]. Traditionally, surgical fields like ophthalmology have been male-dominated, but recently, there has been a greater acceptance and accommodation of women in these specialties. Our results show that given equal opportunities and education, female medical students today have an equal interest in pursuing surgical careers as their male counterparts; it is like what has been reported in a previous local study [3]. 

Among the two most common overall factors that drive the students towards ophthalmology in our study were fewer working hours and the potential for life-work balance; these results are consistent with the most common factor among medical graduates internationally [12], in comparison to one local study [8], which showed the personal interest was the most common factor leading to choose ophthalmology as a career among local medical graduates, this difference may be explained by not specifying what personal causes led the students to prefer ophthalmology.

Regarding the factors that led to not choosing ophthalmology, being already passionate about another specialty was the most common cause for medical students and interns in our study, which is opposing to what has been reported in a study conducted in Saudi Arabia as it showed the ability to have unsatisfied patients and highly competitive specialty were the two most common causes to push the students away from ophthalmology [8].^ ^

However, some limitations should be considered in interpreting these results. Firstly, the study was based on self-reported survey data, which can be subject to response and recall bias. Participants may not have responded with their true interests or may have misremembered past experiences.

## Conclusions

In conclusion, we found that gender did not significantly impact interest in an ophthalmology career among medical students and interns in Saudi Arabia. Females were as likely as males to express a desire to pursue ophthalmology. However, future research should incorporate a broader range of influences on career choice to gain a fuller understanding of factors driving interest in ophthalmology and other specialties. Addressing these additional factors may help in recruitment and ensure the growth of ophthalmology.
